# Cholesteric Molecular Tweezer Artificial Receptor for Rapid and Highly Selective Detection of Ag^+^ in Food Samples

**DOI:** 10.3390/molecules26226919

**Published:** 2021-11-17

**Authors:** Zhe Liu, Ying Ye, Hong Wang, Li-xia Luo

**Affiliations:** 1 College of Agriculture and Animal Husbandry, Qinghai University, Xining 810016, China; lz239880356@163.com (Z.L.); luolixia0515@163.com (L.-x.L.); 2 College of Chemical Engineering, Qinghai University, Xining 810016, China; whong714@126.com

**Keywords:** cholesteric molecular tweezer, artificial receptor, silver ions, naked-eye detection, recognition mechanism

## Abstract

Chiral cholesteric molecular tweezer **7a** was synthesized, and its recognition properties for Ag^+^, Al^3+^, Ca^2+^ etc., were investigated by UV and fluorescence spectra. The results showed that in ethanol/Tris (1/1, *v*/*v*, pH 7.0) buffer solution, the host molecular tweezer **7a** had a specific recognition ability for Ag^+^, the detection limit was up to 1 × 10^−6^ mol/L, and other metal ions had little effect on Ag^+^ recognition. At the same time, the naked-eye detection of Ag^+^ was realized by the light red color of the complex solution. Furthermore, the mechanism of recognition of Ag^+^ by molecular tweezer **7a** was studied by a nuclear magnetic titration test and computer molecular simulation, and a rapid detection method of Ag^+^ using host molecular tweezer **7a** was established. Through the determination of Ag^+^ in milk powder, quinoa and other food samples, it was proved that this novel method had a good application prospect for the detection of Ag^+^ in food.

## 1. Introduction

As is well known, metal ions widely exist in our daily life, especially in the fields of food, the environment, medical treatment etc. However, due to the insufficient prevention of the negative effects brought about by rapid economic and social development, a large number of untreated toxic and harmful ions are indiscriminately released into the environment and enter the human body through the food chain, endangering human health [1,2]. Silver, a transition metal, is one of the most important micronutrients in nature and is now widely used in photography and semiconductor materials due to its optical properties [3,4]. Scientific research reveals that a certain concentration of silver ions shows a good bactericidal effect [5], and not only can it be used for the production of disinfectants [6], but it can also be successfully applied for the prevention and treatment of some diseases and the development of antimicrobial drugs [7]. Although Ag^+^ has shown beneficial effects, once it is continuously accumulated in the body in excess of the safe concentration, it will interact with sulfhydryl groups in various metabolites to inactivate sulfur-containing enzymes [8], thus causing serious adverse effects on human health, such as inhibiting cell proliferation and differentiation [9] and causing skin tissue damage [10], liver and kidney failure and mitochondrial dysfunction [11,12]. Therefore, the development of a highly sensitive silver ion recognition receptor and a reliable detection method is of great significance for human health and food safety. At present, the detection of silver ions is usually limited to traditional analytical methods, including atomic absorption spectroscopy (AAS) [13,14], atomic emission spectroscopy (AES) [15,16], inductively coupled plasma mass spectrometry (ICP-MS) and the ion selective electrode method (ISE) [17,18,19,20]. However, these methods not only rely on expensive large-scale precision instruments, cumbersome pretreatment and skilled professional technicians, but they also have time-consuming operation steps, seriously affecting the efficiency of routine and on-site detection. Nowadays, photochemical technology is gradually developing into a detection method with broad application prospects, which not only has the high selectivity and sensitivity required by detection technology but also needs low-cost instruments and equipment, and the experimental operation is environmentally friendly and efficient, in line with the concept of green chemical detection.

In recent years, many kinds of cationic-specific recognition receptors based on photochemical response signals have been widely reported. Jia et al. [21] developed a benzothiazole-based fluorescence probe for the recognition of Cu^2+^, which could detect Cu^2+^ in acetonitrile solution with high selectivity. Wang et al. [22] prepared dipyrene derivatives from pyrene and pyridine, and the fluorescence experiments showed that the compounds exhibited good selectivity to Ag^+^. Based on coumarin, Chen et al. [23] discovered a highly selective and sensitive fluorescence probe for Pd^2+^. In addition, the cholesteric compounds also showed good selectivity and sensitivity in the recognition fields. Cholesteric compounds have rigid pincer-like structures with rigid molecular skeletons as spacers and aromatic or heterocyclic rings as arms. Different types of molecular tweezers were formed by modifying hydroxyl and carboxyl groups in cholesteric compounds, which could provide a good microenvironment for molecular recognition. Nath et al. [24] synthesized a novel steroidal sensor based on the chiral compound deoxycholic acid, which could detect K^+^ with high sensitivity and selectivity in methanol solution. Jian Wang et al. [25] prepared a cholesteric molecular receptor with a chiral center based on α-hyodeoxycholic acid and found that the receptor had good recognition and coordination properties to amino acid methyl esters; the binding constant with L-alanine methyl ester was as high as 5.24 × 10^3^ L/mol. Furthermore, by taking lithocholic acid as raw material, a kind of chiral asymmetric urea molecular tweezer was designed and synthesized by Xingli Liu et al. [26], and the results showed that these receptors could recognize the guest anions Cl^−^, Br^−^ and I^−^ and form 1:1 supramolecular complexes between host and guest. Thus, it was concluded that cholesteric molecular tweezers have good recognition performance, due to their flexible and multifunctional binding sites, and could be applied for the rapid detection of chemical components in environmental analysis, food analysis etc., as a probe.

In this study, under microwave radiation, a cholesteric molecular tweezer artificial receptor was synthesized via a series of chemical reactions using deoxycholic acid as raw material. Through UV absorption spectroscopy and fluorescence spectroscopy, the selective recognition abilities of different metal ions by the molecular tweezer artificial receptor **7a** were investigated. In addition, a rapid and sensitive method for detecting silver ions using the molecular tweezer **7a** was established; we could even recognize silver ions by the color changing of solutions. Furthermore, the artificial receptor **7a** was successfully applied for the determination of silver ions in some food samples.

## 2. Results and Discussion

### 2.1. The Selectivity of Molecular Tweezer ***7a*** to Ag^+^

**Figure 1:**
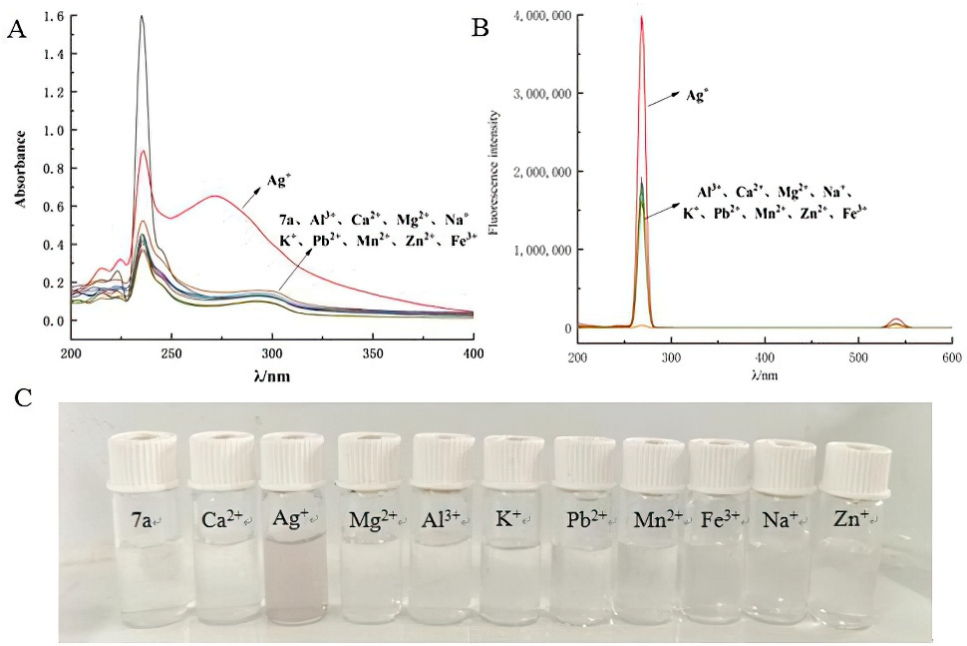
(**A**) UV-Vis absorption spectra of compound **7a** (1.6 × 10^−^^5^ mol/L) with the addition of different metal ions (8.3 × 10^−5^ mol/L). (**B**) Fluorescence spectra of host compound **7a** (1.6 × 10^−^^5^ mol/L) after adding different metal ions (8.3 × 10^−^^5^ mol/L). (**C**) Color change of host compound **7a** (1.6 × 10^−^^5^ mol/L) with different metal ions (8.3 × 10^−^^5^ mol/L).

As shown in Figure 1A, the molecular tweezer **7a** had a strong UV absorption peak at 235 nm. When different metal ions were added into the solution of the molecular tweezer **7a** separately, the absorbance band at 235 nm weakened, especially when Ag^+^ was added. The color of the solution changed from colorless to light red (Figure 1C), and the shape of the UV absorption peak also changed; a new absorption peak even appeared at 270 nm. It is speculated that the interaction between **7a** and Ag^+^ resulted in the change in the spatial structure of **7a** and the formation of the complex and that, then, the new absorption peak appeared and caused the change in the solution color. The fluorescence spectra of the host compound **7a** with different metal ions are shown in Figure 1B. The fluorescence intensity of the host **7a** solution was enhanced after the addition of metal ions, especially after adding Ag^+^, and the fluorescence intensity was more than twice as strong as that of the other cations, all of which revealed that the compound **7a** had excellent selective recognition for Ag^+^ and that the presence of Ag^+^ could be detected by the solution changing color. This behavior may be explained by the fact that the size of the cavity in **7a** matches silver ions and is suitable for silver ions to enter.

### 2.2. Influence of Coexisting Ions and pH Value on the Recognition of Ag^+^ by Probe ***7a***

**Figure 2:**
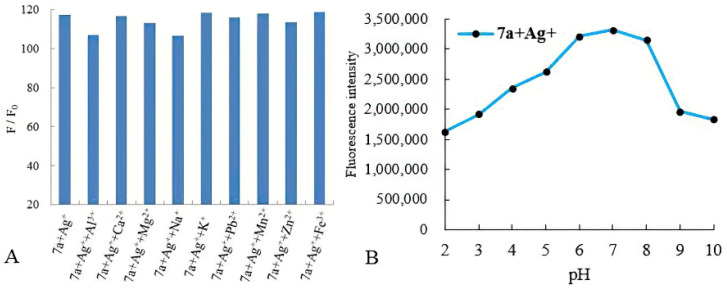
(**A**) Effect of other metal ions (4.5 × 10^−^^5^ mol/L) on fluorescence intensity of host **7a** (9 × 10^−^^6^ mol/L) for Ag^+^ (4.5 × 10^−^^5^ mol/L) recognition. (**B**) Fluorescence spectra of the host compound **7a** (9 × 10^−^^6^ mol/L) for Ag^+^ (4.5 × 10^−^^5^ mol/L) recognition in different pH values.

In order to investigate the influence of other metal ions on the recognition of Ag^+^ by the host compound **7a**, an interference test was carried out, and mixed host (**7a**)–guest (Ag^+^) systems with different pH values were prepared to observe the effect of pH value on the recognition process. As can be seen in Figure 2A, in the system with the co-existence of probe **7a** and Ag^+^, adding five times the amount of other metal ions (Ag^+^, Al^3+^, Ca^2+^, Mg^2+^, Na^+^, K^+^, Pb^2+^, Mn^2+^, Zn^2+^ and Fe^3+^) caused little change in fluorescence intensity compared with adding Ag^+^ only. The relative error values of all the interfering metal ions were found to be less than ± 10% (Table 1) [27], which illustrated that other cations had little effect on the recognition of Ag^+^ by probe **7a**. The effect of pH value on the identification process is displayed in Figure 2B. As can be seen from Figure 2B, the fluorescence intensity increased with the increase in pH. When the pH was about 7, the fluorescence response of the complex tended to be stable, and the intensity gradually reached the maximum. It was speculated that the reason for this might be the effect of hyperacidity or hyperalkalinity on the spatial structure of the molecular tweezer **7a**, which could lead to a change in the size of the molecular cavities and destroy the recognition force, thus resulting in a change in the recognition ability with Ag^+^. The spatial structure of the complexes changed, and then, the fluorescence intensity fluctuated significantly. Therefore, the ethanol/Tris solution with pH 7.0 was chosen as the solvent for the recognition system [28].

### 2.3. UV–Vis Spectral Titration Test and Job’s Plot Experiment

**Figure 3:**
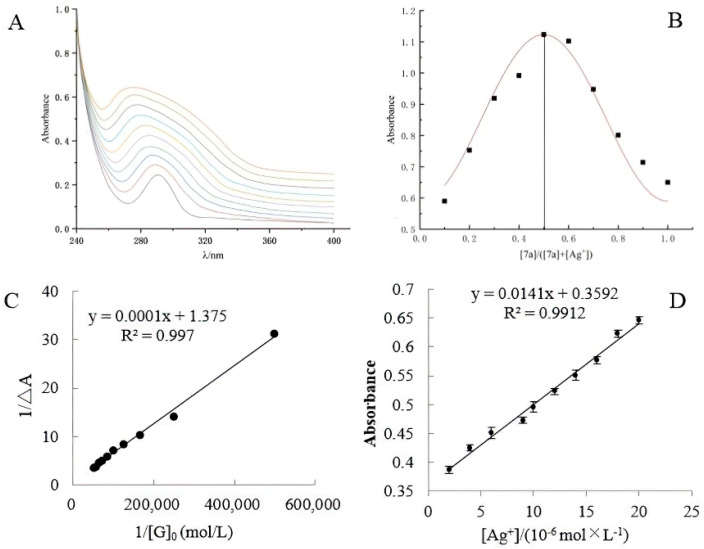
(**A**) UV-Vis spectra of the compound **7a** (1.0 × 10^−4^ mol/L) in the presence of different concentrations of Ag^+^ (0–20 μmol/L). (**B**) Job’s plot for the inclusion complex of host compound **7a** with Ag^+^. (**C**) Typical plot of 1/[G]_0_ versus 1/ΔA of the complex formed by the host compound **7a** and Ag^+^. (**D**) The linear plot of absorbance against the concentration of Ag^+^ (2–20 μmol/L).

A UV–Vis spectral titration test and Job’s plot experiment were carried out to study the recognition abilities of probe **7a** for Ag^+^, and the analytical parameters of the two experiments are displayed in Table 2. In the UV–Vis spectral titration test, when Ag^+^ (0–20 μmol/L) was added into the solution of probe **7a**, the absorbance band at 290 nm consistently increased and shifted to 270 nm (Figure 3A). According to the Hildebrand–Benesi equation [29], the plots of 1/[G]_0_ versus 1/ΔA gave a straight line (Figure 3C), which indicated that probe **7a** could form a complex with Ag^+^ and that the complex consisted of 1:1 host (**7a**) and guest (Ag^+^). According to the intercept and the slope of the straight line, the binding constant Ka of the complex was 1.38 *×* 10^4^ L/mol. The results of the Job’s plot experiment are presented in Figure 3B. When the ratio of [Ag^+^] to [**7a** + Ag^+^] was 0.5, the maximum UV–Vis absorbance was obtained, which also supported the determination of the combination ratio between probe **7a** and Ag^+^ being 1:1.

The linearity and detection limit of the method using probe **7a** to detect Ag^+^ were investigated. As shown in Figure 3A, when different concentrations of Ag^+^ were added into the solution of compound **7a**, the UV-Vis absorbance value of the complex **7a**+Ag^+^ consistently increased. Taking the concentration of Ag^+^ as abscissa and the UV absorbance as ordinate, a straight line was obtained in the linear range of 2 × 10^−6^ to 2 × 10^−5^ mol/L (Figure 3D), and the linear equation was y = 0.014x + 0.359 (R^2^ = 0.991). According to the formula LOD = 3σ/k [30,31] (k is the slope of the standard curve y = 0.014x + 0.359, and σ is the standard deviation), the limit of detection was calculated to be 1.0 μmol/L, which is superior to most reported probes for detecting Ag^+^ [32,33,34,35,36]; the compared results are presented in Table 3. It is concluded that probe **7a** is clearly more sensitive than most of the other sensors for the detection of Ag^+^ [32,33,34,35,36]. Moreover, this detection system for Ag^+^ is visual, rapid and without complicated process and expensive equipment.

### 2.4. Microstructure Observation and Energy Spectrum Image Analysis

**Figure 4:**
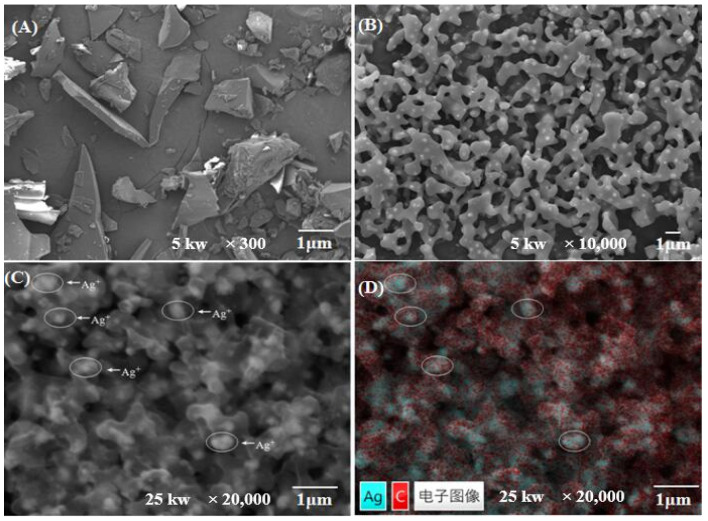
(**A**) FESEM image (×300, 5 kw) of compound **7a** in the absence of Ag^+^. (**B**) FESEM image (×10,000, 5 kw) of compound **7a** in the presence of Ag^+^. (**C**) FESEM image (×20,000, 25 kw) of compound **7a** in the presence of Ag^+^. (**D**) Energy spectrum image (×20,000, 25 kw) of compound **7a** in the presence of Ag^+^.

The field-emission scanning electron micrograph (FESEM) of the complex formed by probe **7a** and Ag^+^ is presented in Figure 4. As it can be seen, the compound **7a** was mainly packed in a dense block form (Figure 4A), and when it was complexed with Ag^+^ (Figure 4B), the structure of compound **7a** obviously changed, forming a regular network structure with a large number of cavities, and silver ions were embedded in them, forming stable molecular aggregates [37]. In order to confirm that the cavities as shown in the figure were embedded with Ag^+^, the JSM 7900F high-resolution transmission scanning electron microscope was used to take a scanning electron microscope image (Figure 4C) and a corresponding energy spectral image (Figure 4D) of the complex compound at an accelerated voltage of 25.0 KV. As demonstrated in Figure 4C, a large number of silver ions entered the clefts of the host molecular tweezer **7a**. Through an analysis of the energy spectrum image, it was found that the blue part in Figure 4D was Ag^+^ and that the red part was the carbon skeleton of the host molecular tweezer **7a**, which proved that most of the silver ions were successfully embedded in the cavities and were encapsulated by compound **7a** to form a stable complex.

### 2.5. Analysis of the Mass Spectra and Infrared Spectra of the Complex

**Figure 5:**
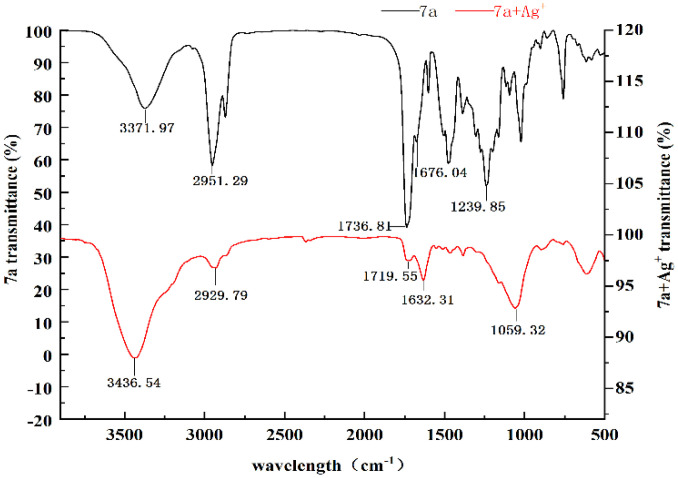
Infrared absorption spectra of compound **7a** and complex compound **7a** + Ag^+^.

The mass of compound **7a** was m/z 755.33, and the measured mass of complex **7a** + Ag^+^ was m/z 864.11, which was consistent with the theoretical value m/z 864.20 (Figure S1, Supplementary Materials), further confirming that the probe **7a** formed a complex with Ag^+^. The infrared spectra of the host compound **7a** and the complex **7a** + Ag^+^ are shown in Figure 5. As can be seen from the infrared spectra of compound **7a**, the absorption band at 3372 cm^−1^ was assigned to the stretching vibrations of amide N-H groups; the absorption peak at 2951 cm^−1^ corresponded to the stretching frequencies of methoxy C-H; the high-intensity peak at 1737 cm^−1^ was related to the stretching vibrations of the carbonyl group; and the absorption band at 1239 cm^−1^ was ascribed to C-O-C symmetric stretching vibrations. After compound **7a** binding with Ag^+^, the vibration absorption peak of amide N-H at 3372 cm^−1^ shifted to 3437 cm^−1^, and the stretching peak of methoxy C-H at 2951 cm^−1^ changed to 2930 cm^−1^; the characteristic absorption peak of C=O moved from 1737 cm^−1^ to 1720 cm^−1^, and the stretching absorption peak of C-O-C of the methoxy group shifted from 1239 cm^−1^ to 1059 cm^−1^; furthermore, the peak shape at 2951 cm^−1^ and 1737 cm^−1^ changed a lot, all of which indicated that C=O and -OCH_3_ might play a critical role in coordination with Ag^+^. Based on the infrared spectra, it was inferred that Ag^+^ lacked electrons, while C=O and -OCH_3_ provided electrons to it, which led to electric charge transfer and, thus, caused the change in the infrared spectrum of compound **7a**.

### 2.6. Results and Analysis of Nuclear Magnetic Titration Test

**Figure 6:**
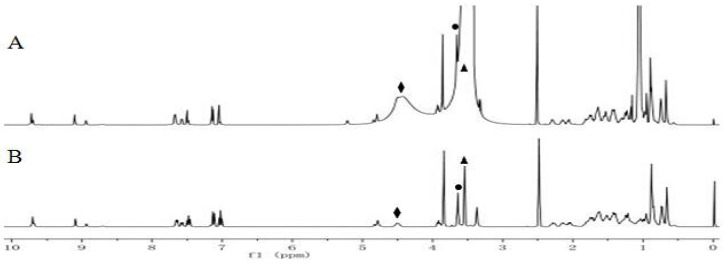
Portion of ^1^H NMR spectra of compound **7a** in DMSO-d_6_ in the presence of increasing amount of Ag^+^: (**A**) +1 equiv; (**B**) +0 equiv; (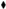, NCH; ▴, 12α-COOCH_3_; •, ArOCH_3_).

In order to further study the recognition mechanism of molecular tweezer **7a** and Ag^+^, a nuclear magnetic titration test was performed. The ^1^H NMR spectra are presented in Figure 6, from which it can be seen that compound **7a** displayed a single peak at 4.52 ppm (Figure 6B), which was assigned to -NCH and belonged to the amino acid structure, and two other sharp signals were found at 3.53 and 3.62 ppm, which belonged to COOCH_3_ in the 12α-moiety and OCH_3_ attached to the phenyl ring, respectively. When Ag^+^ at 1.0 equiv was added into the solution of compound **7a** (Figure 6A), the proton signal peak at 4.52 ppm downfield shifted to 0.1ppm; in particular, the signal peak at 3.53 ppm belonging to COOCH_3_ in the 12α-moiety became significantly wider and stronger, which almost overlapped the peak at 3.62 ppm. It was speculated that the oxygen atoms in the 12α-COOCH_3_ and Ar-OCH_3_ groups provided electrons to Ag^+^, which resulted in the change in the neighboring proton signal peaks.

### 2.7. Computer Molecular Simulation

**Figure 7:**
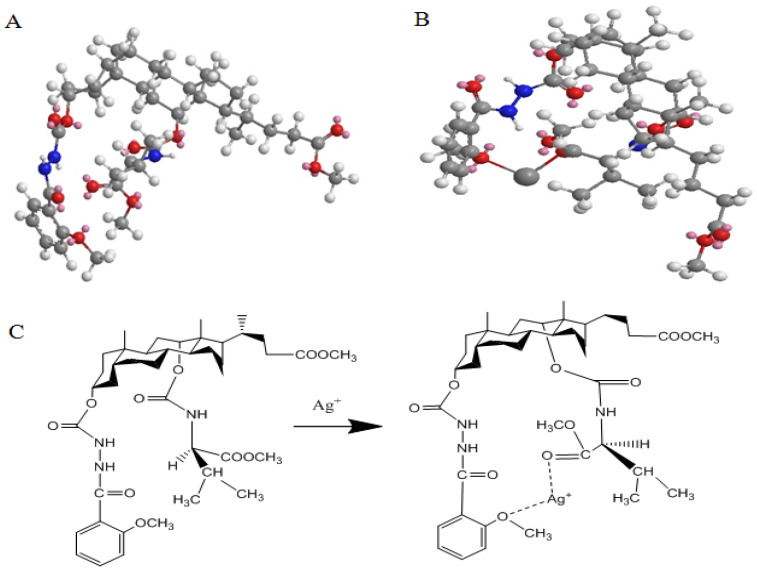
(**A**) The energy-minimized conformation of molecular tweezer **7a**. (**B**) The energy-minimized conformation of complex compound **7a** + Ag^+^. (**C**) Possible binding model of molecular tweezer **7a** and Ag^+^.

The computer molecular modeling software (Chem 3D) was applied to systematically search the conformation of the compounds [38]. The minimum energy molecular conformations of probe **7a** and the complex **7a** + Ag^+^ are displayed in Figure 7. When it was in the minimum energy conformation, probe **7a** presented a pincer shape, which provided enough space for the insertion of Ag^+^. In the formation of the complex compound **7a** + Ag^+^, the shortest distance between the oxygen atom in the Ar-OCH_3_ group and Ag^+^ was 2.028 Å, and the shortest distance between the oxygen atom in the carbonyl group (12α-COOCH_3_) and Ag^+^ was 1.994 Å. It was suggested that the oxygen atoms in the 12α-COOCH_3_ and Ar-OCH_3_ groups interacted with Ag^+^ to form a complex and that the possible recognition driving forces could be hydrogen bonds and electrostatic attraction. The possible binding model of molecular tweezer **7a** with Ag^+^ is presented in Figure 7C.

### 2.8. Quantitative Determination of Ag^+^ in Food Samples

The application of the molecular tweezer **7a** for Ag^+^ sensing in different food samples was investigated. The recoveries of this method were obtained from the ratio of the measured value of the average Ag^+^ content in the sample determined three times to the theoretical value by adding a series of different known concentrations of Ag^+^ into the sample. As shown in Table 4, the amounts of Ag^+^ in some food samples, namely, milk powder, wheat flour, quinoa and wolfberry, detected by the molecular tweezer **7a**, were 19.44, 31.06, 41.40 and 38.37 μg/g, respectively, compared with the AAS method; the relative error was less than 5%. Quantitative spike recoveries for the detection of Ag^+^ in these samples ranged from 99% to 104% (Table 5), with a relative standard deviation of less than 3% (*n* = 3), which further demonstrated that the molecular tweezer **7a** was reliable for monitoring Ag^+^ in food samples.

## 3. Materials and Methods

### 3.1. Chemical Reagents and Instruments

All the reagents and solvents used for synthesis were analytically pure and used without further treatment unless otherwise noted. A Tris-HCl buffer solution (pH 7.4) was prepared using 0.1 mol/L HCl and proper amount of a 0.1 mol/L Tris stock solution (Sinopharm Chemical Reagent Company, Shanghai, China). Double-distilled water was used throughout the process of solution preparation and spectroscopic testing. The solutions of the metal ions were prepared from their nitrate salts. Different food samples, namely, milk powder, wolfberry, quinoa and wheat flour, were bought from the local market (Xining, China).

Chemical reactions were conducted by MCR-3 microwave chemical reactor (Zhengzhou, China). Ultraviolet spectra were measured by UV-2600 Shimadzu ultraviolet–visible spectrophotometer (Kyoto, Japan). Fluorescence spectra were recorded by RF-6000 Shimadzu fluorescence spectrophotometer (Kyoto, Japan). Mass spectra were analyzed by Thermo Q Exactive ultra-high-resolution liquid chromatography–mass spectrometer (Shanghai, China). The ^1^H-NMR spectra were recorded with the AVANCE NEO nuclear magnetic resonance spectrometer (Rheinstetten, Germany). The microscopic images were observed with the JSM-7900F field-emission scanning electron microscope (Kyoto, Japan). The infrared spectra were recorded with the Nicolet 6700 Fourier transform infrared spectrometer (Shanghai, China).

### 3.2. Preparation of Cholesteric Molecular Tweezer Artificial Receptor ***7a***

**Scheme 1:**
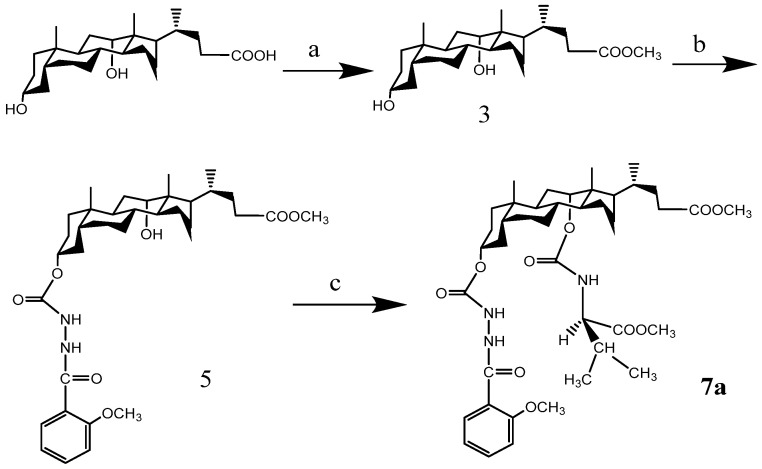
The synthetic route of molecular tweezer **7a**. Reagents and conditions: (**a**) CH_3_OH, H_2_SO_4_; (**b**) N_2_H_4_•H_2_O, triphosgene, CH_2_Cl_2_, MWI; (**c**) triphosgene, CH_2_Cl_2_, pyridine, MWI; L-valine acid methyl ester hydrochloride, pyridine, MWI.

Cholesteric molecular tweezer **7a** was synthesized according to the method reported in Reference [39]. The intermediate 5 (0.5 mmol/L), anhydrous dichloromethane (10 mL), anhydrous pyridine (0.5 mL) and triphosgene (0.18 mmol/L) were placed in a 50 mL flask and heated under 300 W microwave conditions for 10 min. When the reaction was complete, valine methyl ester hydrochloride (1 mmol/L) and anhydrous pyridine (0.5 mL) were added, and the reaction continued for 10 min under the same conditions. After the reaction was complete, the solvent was evaporated under vacuum, and the residue was purified by column chromatography to obtain molecular tweezer **7a** (C_41_H_61_N_3_O_10_). The synthesis route is shown in Scheme 1. The mass spectrum, ^1^H-NMR and ^13^C-NMR spectra of the target compound **7a** were consistent with what was reported in the literature [40]. The synthesis route of molecular tweezer **7a** is shown in Scheme 1.

### 3.3. General Procedure

Compound **7a** was dissolved in 5 mL of DMSO, then ethanol/Tris (*v*/*v* = 1/1, pH 7.0) was added to a certain volume, and then a molecular tweezer reserve solution of 0.1 mmol/L was obtained. Nitrate and acetate of cations (Ag^+^, Al^3+^, Ca^2+^, Mg^2+^, Na^+^, K^+^, Pb^2+^, Mn^2+^, Zn^2+^, Fe^3+^) were dissolved in ultra-pure water, and then 0.1 mmol/L stock solutions of cations were prepared [41]. At room temperature, a certain amount of different metal ions were added to the molecular tweezer reserve solution, and then the UV-Vis absorption spectra and the fluorescence spectra of the mixture were measured (selectivity test, interference test, UV-Vis spectral titration test and Job’s plot experiment). The fluorescence spectra were measured in the range of 200–600 nm, and the ultraviolet absorption spectra were measured in the range of 200–400 nm.

In the selectivity test, 0.5 mL of host molecular tweezer **7a** with a concentration of 1 × 10^−4^ mol/L was added with 5 times the amount of metal ions, and then, by observing the changes of the solution color and the UV–Vis absorption spectra, the selective recognition abilities of molecular tweezer **7a** for various metal ions were determined.

In the UV-Vis spectral titration test, Ag^+^ (0–20 μmol/L) was gradually dropped into the host molecular tweezer **7a** solution with a concentration of 1.0 × 10^−4^ mol/L. The UV-Vis absorbance values of the complex solution were measured at different Ag^+^ concentrations, and according to the Hildebrand-Benes equation [29], the complexation constant of the host molecular tweezer **7a** and Ag^+^ was calculated.

In the Job’s plot experiment, a series of mixed solutions containing 1:9, 2:8, 3:7, 4:6, 5:5, 6:4, 7:3, 8:2 and 9:1 of the concentration ratio of the host molecular tweezer **7a** to the guest Ag^+^ was prepared, and their absorbance values were determined. The Job curve of the complex was obtained by mapping the molar ratio of host to guest, and the combination ratio of host molecular tweezer **7a** and the guest Ag^+^ was determined [42].

### 3.4. Determination of Ag^+^ in Food Samples

Two grams of samples of milk powder, wolfberry, quinoa and wheat flour was taken, dried, crushed and then transferred into a digestion tube. Then, 0.2 g of copper sulfate, 6 g of potassium sulfate and 10 mL of sulfuric acid were added successively. The samples were digested at 400 °C for two hours, cooled to room temperature, diluted to a certain multiple and adjusted to pH 7.0. Then, 5 mL of the host molecular tweezer **7a** (1 × 10^−^^4^ mol/L) was added into the test tube, and 2.5 mL of the diluted samples were mixed. The absorbance of the mixture at 270 nm was measured. The mean value of each group was determined three times, and the Ag^+^ content in each sample was calculated according to the standard curve regression equation.

## 4. Conclusions

Taking deoxycholic acid as material, molecular tweezer **7a** was synthesized. Using ultraviolet spectrophotometry and fluorescence titration, we found that the addition of Ag^+^ could cause regular changes in the UV and fluorescence spectra of molecular tweezer **7a**, and the addition of other metal ions had little interference, which indicated that molecular tweezer **7a** had high selectivity for Ag^+^. The binding constant of **7a** + Ag^+^ was 1.38 × 10^4^ L/mol, and the detection limit was 1 μmol/l, higher than that of many reported methods [32,33,34,35,36]. In the ethanol/Tris solution (*v*/*v* = 1/1, pH = 7.0), adding Ag^+^ changed the color of the molecular tweezer **7a** solution from colorless to light red, while the color of the solutions with other metal ions added did not change; thus, it was easy for us to realize the rapid naked-eye detection of Ag^+^. In addition, the combination of Ag^+^ with molecular tweezer **7a** was further examined by Job’s method, and the coordination ratio of Ag^+^ with **7a** was found to be 1:1. In order to further investigate the mechanism of the recognition between molecular tweezer **7a** and Ag^+^, the changes in IR, MS and ^1^H NMR before and after the combination of molecular tweezer **7a** and Ag^+^ were studied. It was found that Ag^+^ could enter the clefts of molecular tweezer **7a**, groups 12α-COOCH_3_ and ArOCH_3_ in the molecular tweezer **7a** were the main binding sites, and the oxygen atoms in the groups could provide electrons for Ag^+^. The recognition driving forces might be electrostatic gravitation and hydrogen bonding. Furthermore, molecular tweezer **7a** was applied for the determination of Ag^+^ in real food samples, and a method for the determination of Ag^+^ in real food samples was established by the determination of Ag^+^ in quinoa, wheat flour etc., and compared with the method of AAS. The new method is low cost with simple operation, and it is also reliable; therefore, it is expected to be developed as a rapid detection kit for Ag^+^ detection in food, the environment and other areas.

In summary, we successfully demonstrated a cholesteric molecular tweezer artificial receptor for Ag^+^ detection in food samples. Compared with other methods that have been reported, this method exhibits some fascinating properties, namely, excellent selectivity, high sensitivity, a wide linear range, cost effectiveness and the ability to visually detect Ag^+^. Notably, the detection system was successfully used for the analysis of Ag^+^ in some dairy products, grains and berries, indicating that this novel method is useful in actual food samples. We believe that such a cholesteric molecular tweezer artificial receptor will have great potential applications for Ag^+^ determination in food analysis, environmental monitoring, medicine and other fields.

## Figures and Tables

**Table 1:** Effect of 9 metal ions on the determination of Ag^+^ (Ag^+^ = 4.5 × 10^−^^5^ mol/L).

Metal Ions	Concentration (mol/L)	Relative Error/%	Metal Ions	Concentration (mol/L)	Relative Error/%
Al^3+^	4.5 × 10^−5^	9.5	Mn^2+^	4.5 × 10^−5^	−0.8
Ca^2+^	4.5 × 10^−5^	0.6	Zn^2+^	4.5 × 10^−5^	3.1
Mg^2+^	4.5 × 10^−5^	3.5	Fe^3+^	4.5 × 10^−5^	−1.2
Na^+^	4.5 × 10^−5^	9.7	Pb^2+^	4.5 × 10^−5^	1.1
K^+^	4.5 × 10^−5^	−1			

**Table 2:** The analytical parameters of the UV–Vis spectral titration test and Job’s plot experiment.

Compound	Absorption Coefficient (L/mol·cm^−1^)	Linear Dynamic Range (mol/L)	Light Path Length of Sample Pool (cm)	Maximum Wavelength (nm)	Association Constant (L/mol)
**7a** + Ag^+^	0.039 × 10^−6^	2 × 10^−6^ ~ 2 × 10^−5^	1	270	1.38 × 10^4^

**Table 3:** Comparison of various recognition sensors for Ag^+^.

Compound	Solvent	Detection Limit	Ref.
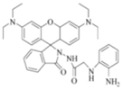	Water	1.25 × 10^−5^ M	[32]
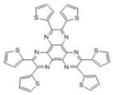	Chloroform	1.79 × 10^−6^ M	[33]
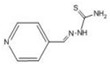	CH_3_OH/Tris-HCl	1.6 × 10^−6^ M	[34]
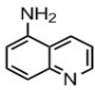	H_2_O	5.3 × 10^−5^ M	[35]
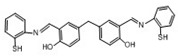	DMSO/H_2_O	6.37 × 10^−5^ M	[36]
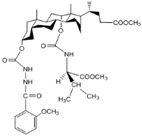	Tris/HCl	1 × 10^−6^ M	This work

**Table 4:** Comparison of new method and AAS method for Ag^+^ determination in food samples (*n* = 3).

Samples	New Method μg/g	RSD (%)	AAS Method μg/g	Relative Error (%)
milk powder	19.44 ± 0.28	1.43	18.65 ± 0.80	4.24
wheat flour	31.06 ± 0.70	2.25	32.00 ± 0.38	−2.93
quinoa	41.40 ± 0.47	1.14	40.20 ± 0.75	2.99
wolfberry	38.37 ± 0.27	0.72	37.58 ± 0.56	2.11

**Table 5:** Recovery of Ag^+^ in different food samples (*n* = 3).

Samples	Add (μg/g)	This Method (μg/g)	Recovery (%)	RSD (%, *n* = 3)	AAS Method (μg/g)	Recovery (%)	RSD (%, *n* = 3)
quinoa	10	51.52 ± 0.13	99	2.46	52.62 ± 0.20	103	2.29
	40	85.33 ± 0.11	104	1.34	88.77 ± 0.20	109	3.86
wolfberry	10	50.85 ± 0.12	103	2.35	46.75 ± 0.07	97	1.66
	40	81.76 ± 0.20	104	2.50	74.23 ± 0.34	95	4.59

## Data Availability

The data presented in this study are available in the article and Supplementary Materials.

## Supplementary Materials

The following are available online. Figure S1: ESI-MS spectrum of complex compound **7a** + Ag^+^.

## Sample Availability

Samples of compounds **7a**(3α-[o-methoxy-1-(N-formyloxyamino)] benzoylhydrazine-12α-[2-(N-formyloxyamino)-3-methyl]butyrate methyl ester-5β-24-cholic acid methyl ester) are available from the authors.
