# Supplementary material for: Cholesteric Molecular Tweezer Artificial Receptor for Rapid and Highly Selective Detection of Ag+ in Food Samples
Source: Molecules. 2021 Nov 17;26(22):6919. doi: 10.3390/molecules26226919 (PMC8617623; doi:10.3390/molecules26226919)
Supplement: Supplementary file 1 [file molecules-26-06919-s001.zip › molecules-1410809-supplementary.pdf]

Cholesteric Molecular Tweezer Artificial Receptor for Rapid and Highly Selective Detection of Ag^+ in Food Samples

Zhe Liu [1], Ying Ye [1]*, Hong Wang[2] and Li-xia Luo[1]

1 College of Agriculture and Animal Husbandry, Qinghai University, Xining, 810016 China; lz239880356@163.com (Z.L.); luolixia0515@163.com (L.-x.L.)
2 College of Chemical Engineering, Qinghai University, Xining 810016, China; whong714@126.com
* Correspondence: yeying08211983@163.com

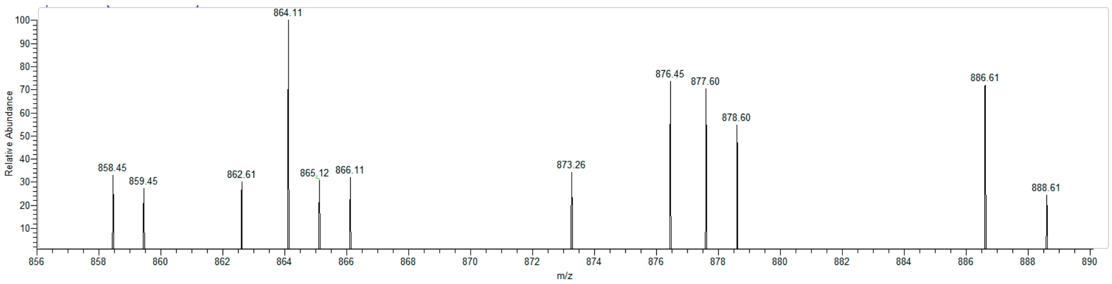

Figure S1. ESI-MS spectrum of complex compound 7a+Ag^+.
